# *Skopi*: a simulation package for diffractive imaging of noncrystalline biomolecules

**DOI:** 10.1107/S1600576722005994

**Published:** 2022-07-15

**Authors:** Ariana Peck, Hsing-Yin Chang, Antoine Dujardin, Deeban Ramalingam, Monarin Uervirojnangkoorn, Zhaoyou Wang, Adrian Mancuso, Frédéric Poitevin, Chun Hong Yoon

**Affiliations:** a Linac Coherent Light Source, SLAC National Accelerator Laboratory, 2575 Sand Hill Road, Menlo Park, CA 94025, USA; b European XFEL, Holzkoppel 4, 22869 Schenefeld, Germany; c Department of Chemistry and Physics, La Trobe Institute for Molecular Science, La Trobe University, Melbourne, Victoria 3086, Australia; DESY, Hamburg, Germany

**Keywords:** single-particle imaging, fluctuation X-ray scattering, holography, free-electron lasers, simulation

## Abstract

X-ray free-electron lasers (XFELs) have the ability to produce ultra-bright femtosecond X-ray pulses for coherent diffraction imaging of biomolecules. While the development of methods and algorithms for macromolecular crystallography is now mature, XFEL experiments involving aerosolized or solvated biomolecular samples offer new challenges in terms of both experimental design and data processing. *Skopi* is a simulation package that can generate single-hit diffraction images for reconstruction algorithms, multi-hit diffraction images of aggregated particles for training machine learning classifiers using labeled data, diffraction images of randomly distributed particles for fluctuation X-ray scattering algorithms, and diffraction images of reference and target particles for holographic reconstruction algorithms. *Skopi* is a resource to aid feasibility studies and advance the development of algorithms for noncrystalline experiments at XFEL facilities.

## Abstract 2

The *Skopi* software package provides tools to generate realistic simulations of coherent X-ray diffractive imaging of noncrystalline biological samples, which in turn will aid algorithm development for a range of experiments at X-ray free-electron laser sources.

## 1. Introduction

The unique capabilities of X-ray free-electron laser (XFEL) sources have led to significant advances in structural biology since the first hard X-ray laser began operation at the Linac Coherent Light Source (LCLS) in 2009 ▸ (Emma *et al.*, 2010 ▸; Jamison, 2010 ▸). The XFEL technique of serial femtosecond crystallography has proven particularly transformative for protein crystallographers (Chapman *et al.*, 2011 ▸; Boutet *et al.*, 2012 ▸; Spence, 2018 ▸). Ultra-bright X-rays enable the study of crystals too small or radiation sensitive for synchrotron sources, while the femtosecond pulses have permitted time-resolved studies of enzyme catalysis at atomic resolution (Schlichting, 2015 ▸; Stauch & Cherezov, 2018 ▸). By contrast, coherent X-ray diffractive imaging (CXDI) of noncrystalline biological samples has remained a largely nascent application, despite these experiments being the original intent of XFEL technology (Neutze *et al.*, 2000 ▸; Bogan *et al.*, 2008 ▸). Several studies have demonstrated successful single-particle imaging (SPI) of large targets such as viruses and organelles (Seibert *et al.*, 2011 ▸; van der Schot *et al.*, 2015 ▸; Brändén *et al.*, 2019 ▸; Hantke *et al.*, 2014 ▸), but 3D reconstruction has been limited to nanoscale resolution (Starodub *et al.*, 2012 ▸; Ekeberg *et al.*, 2015 ▸; Rose *et al.*, 2018 ▸; Kurta *et al.*, 2017 ▸). Multi-particle techniques, including fluctuation X-ray scattering (FXS) (Mendez *et al.*, 2014 ▸, 2016 ▸; Kurta *et al.*, 2017 ▸; Doniach, 2018 ▸; Pande *et al.*, 2018 ▸) and Fourier transform holography (FTH) (Eisebitt *et al.*, 2004 ▸; Marchesini *et al.*, 2008 ▸; Shintake, 2008 ▸; Gorkhover *et al.*, 2018 ▸), have also been explored for structure determination. However, CXDI of noncrystalline biological samples remains far from routine, in contrast to the success of XFEL experiments on inorganic materials that provide significantly more signal (Ayyer *et al.*, 2020 ▸). Improvements in resolution are needed before these techniques can address novel questions in structural biology.

The principal challenges facing CXDI studies of non­crystalline samples are the low signal relative to instrumental background, the low hit rate due to difficulties with sample delivery and reliable hit finding, and the intrinsic heterogeneity of the imaged particles, which limits the resolution (Bielecki *et al.*, 2020 ▸; Daurer *et al.*, 2017 ▸). Improvements in sample delivery and in the dynamic range of detectors have increased the quality of the measured signal (Bielecki *et al.*, 2019 ▸), but both experimental and algorithmic advances are needed to achieve high-resolution reconstructions from these data. In particular, accurate classification is required both to identify the useful single-particle hits from shots of aggregates or no particles and to cluster images on the basis of conformational heterogeneity (Maia *et al.*, 2009 ▸; Yoon *et al.*, 2011 ▸; Reddy *et al.*, 2017 ▸; Cruz-Chú *et al.*, 2021 ▸). In addition, more sophisticated background subtraction methods are needed to isolate the low-intensity particle scattering prior to reconstruction. While methods of performing classification and background removal have been explored and applied to the few experimental data sets available (Assalauova *et al.*, 2020 ▸; Shi *et al.*, 2019 ▸), it is unclear how well these algorithms will generalize to new experiments.

Realistic simulations of CXDI experiments could accelerate the maturation of this field by providing a test bed for advanced data pre-processing and reconstruction algorithms. *Condor* is an existing open-source program already available for CXDI simulations and was created to facilitate planning of experiments at first-generation XFELs (Hantke *et al.*, 2016 ▸). However, the ongoing development of different types of SPI experiments with higher repetition rates and more sophisticated detectors poses new challenges and thus calls for tools capable of simulating these technological advances at scale. Here we present *Skopi*, a software package designed for rapid and high-throughput simulations of SPI, FXS and FTH experiments on modern detectors. Additional features of *Skopi* include the ability to simulate single-particle aggregates to facilitate single-particle classification algorithm development using machine learning (Yoon *et al.*, 2011 ▸; Shi *et al.*, 2019 ▸; Ignatenko *et al.*, 2021 ▸) and the modeling of the solvent shell and conformational heterogeneity inherent in biomolecules. The simulated experiments are highly customizable and readily support modeling the beam characteristics and gain auto-ranging detectors available at LCLS as well. *Skopi* also makes it convenient to include diverse sources of noise, including fluctuating dark noise, beam miscentering, a static sloped background and fluence jitter, all of which can impact reconstruction. We anticipate that *Skopi* will be a useful resource for developing new data processing algorithms, guiding experimental design, and in time aiding on-the-fly feedback during CXDI experiments of noncrystalline biological samples by incorporating live beamline measurements to build a digital twin of the experiment.

## 2. Modular implementation

**Figure 1:**
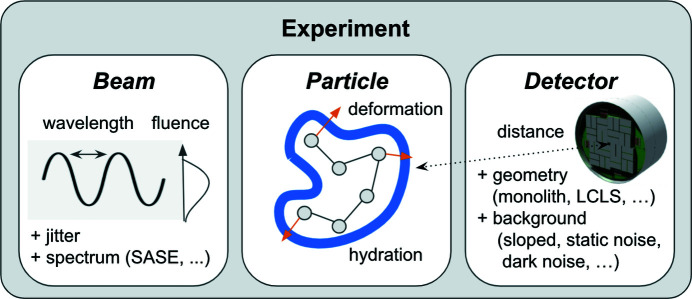
The modular architecture of *Skopi*. The three principal components of each experiment – the beam, particle and detector – are initialized independently of each other. Once these components are set up, diffraction patterns from a range of CXDI experiments can be efficiently simulated.

The design of *Skopi* is highly modular, with the three main components of a CXDI experiment – the particle, beam and detector – agnostic to the type of experiment being simulated (Fig. 1 ▸). Each component can be modeled with a range of complexity, from the ideal case to more sophisticated representations that mimic experimental errors and noise. We briefly describe these components below.

The particle object stores the biomolecule’s atomic coordinates and associated atomic form factors. Coordinates can be supplied as either a PDB or an HDF5 file; the latter format permits coarse-graining by allowing the user to specify ‘atom’ positions and their corresponding form factors. Conformational heterogeneity can be modeled by sampling along the particle’s normal modes to generate alternate states; this is accomplished using the anisotropic network model from the *ProDy* package (Bakan *et al.*, 2011 ▸) [Fig. S1(*a*) in the supporting information]. Although unsuitable for certain types of dynamics like side-chain flips and local unfolding, normal modes analysis offers a general way of modeling large-scale collective motions (Tama & Sanejouand, 2001 ▸). Further, the amplitude of these motions can be tuned by choosing distinct modes and the scale of coordinate deformation along each mode, making it easy to generate data sets with varying amounts of heterogeneity. For types of dynamics not accounted for by normal modes, the user could employ external programs to build a library of discrete conformations, which *Skopi* could then sample from to produce a heterogeneous data set.

Another source of sample heterogeneity encountered in CXDI experiments is the disordered solvent shell surrounding aerosolized particles, which helps to preserve structural integrity in a vacuum but increases shot-to-shot variation (Hau-Riege *et al.*, 2007 ▸; Mandl *et al.*, 2020 ▸). To account for this effect, *Skopi* enables modeling of a hydration layer with a user-defined thickness that follows the particle’s contour [Fig. S1(*b*)]. This solvent mask represents a continuum water model rather than a collection of solvent molecules, and a uniform water density function is sampled at discrete positions within this mask during the diffraction calculation. For particles that contain large cavities, the interior portion of the solvent shell may not entirely fill the particle. In these cases, the remaining void can be modeled using a uniform function of the desired scattering density, which we anticipate will be useful for modeling nucleic acid-containing viruses. A library of particles adopting different conformations or with variable degrees of hydration can be created and then sampled from to generate heterogeneous data sets.

The particle object can be duplicated to form aggregates or clusters. In an experiment, the exact distribution of the number of particles would depend on the particle concentration and parameters governing the injection method (Bielecki *et al.*, 2019 ▸; Welker *et al.*, 2021 ▸). In *Skopi*, we introduce a parameter sticking; when turned on, all the particles in the beam aggregate to form a single cluster. The feature is implemented using a collision model based on the *N*-body simulations of ballistic particle–cluster aggregation (Meakin, 1991 ▸; Okuzumi *et al.*, 2009 ▸), which is a sequence of successive collisions between a cluster and a single particle. The present model uses a ‘hit-and-stick’ collision model, where the particle is randomly rotated and positioned with respect to the cluster and then translated towards the cluster center until they collide. We neglect any effects from compression or fragmentation of the particle during the collision. Fig. S2 illustrates the particle aggregation procedure.

The beam object contains information about the beam’s dimensions, fluence and wavelength spectrum. The simplest case models a single monochromatic spike with spatially uniform fluence. A more advanced option accounts for self-amplified spontaneous emission (SASE), which produces ultra-bright pulses but broadens the beam’s energy spectrum (Geloni *et al.*, 2017 ▸). *Skopi* models SASE spectra as trains of uncorrelated spikes using a Gaussian kernel density estimate to approximate their energy distribution (Fig. S3). Noise can be added by modeling spatial variation of the fluence in the plane of the beam to follow a Gaussian profile and by introducing jitter, in which shot-to-shot changes in total fluence are Gaussian distributed.

The simplest detector is a monolithic square pixel array of user-defined dimensions. The diverse detectors in use at LCLS for CXDI experiments are also supported [Fig. S4(*a*)]. Recently developed detectors, such as the Jungfrau and ePix10k, are implemented with an auto-ranging feature that avoids saturation to increase their dynamic range (Blaj *et al.*, 2015 ▸; van Driel *et al.*, 2020 ▸; Redford *et al.*, 2018 ▸) [Fig. S4(*b*)]. LCLS detectors also have access to the calibration constants and fluctuating dark noise from specific experiments [Fig. S5(*a*)]. Once initialized with a beam, the detector object is populated with information about the pixels’ position in reciprocal space, solid angle subtended and polarization. The detector object also supports modeling various sources of error in addition to fluctuating dark noise. One of these is beam miscentering (Loh *et al.*, 2013 ▸), in which the direct beam position is displaced relative to the detector’s center from shot to shot. These offsets are assumed to be Gaussian distributed and are accounted for by mapping the displaced pixels’ coordinates to their corresponding vectors in reciprocal space [Fig. S5(*b*)]. The user can also supply a custom sloped background to model parasitic scattering or shadows from the experiment’s optics, which are sources of correlated noise that cannot be averaged away by merging more data [Fig. S5(*c*)].

**Figure 2:**
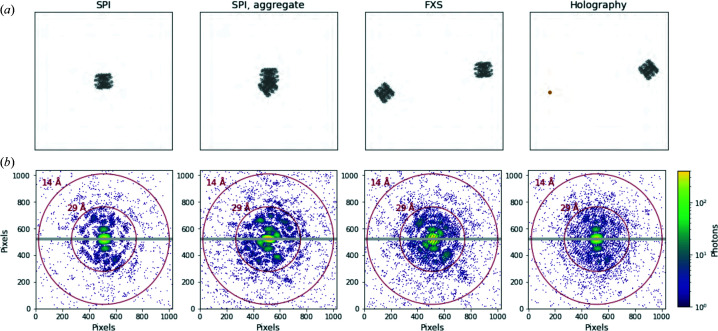
An overview of noncrystalline CXDI experiments. *Skopi* supports simulations of SPI, FXS and FTH experiments. (*a*) Projections of the particle(s) in the plane of the beam and (*b*) the corresponding diffraction patterns, shown for each experiment type. In the case of SPI, either an individual particle or an aggregate can be simulated. In FXS and FTH experiments, multiple particles are in the beam; for FTH, one of these particles serves as a reference, in this case a small cluster of gold atoms. The biomolecule used in these simulations is a chaperonin (PDB 3iyf; Zhang *et al.*, 2010 ▸)). In row (*b*) the gray region marks the gap between panels of the PnCCD detector. The beam fluence has been artificially inflated to aid visualization.

Once the particle, beam and detector are set up, any of the three principal CXDI experiments can be easily and efficiently simulated. *Skopi* provides convenient interfaces for each of the SPI, FXS and FTH experiment types, from which small- or wide-angle X-ray scattering profiles can be derived through radial averaging. In the case of FTH, *Skopi* supports the variation in which the hologram is produced by interference between the target particle and a reference specimen, rather than by modifying the beam shape using structured apertures. For the reference particle, *Skopi* provides a basic gold nanoparticle to facilitate setting up the experiment, while external libraries like the *Atomic Simulation Environment* software package can be employed to generate objects of different shapes (Hjorth Larsen *et al.*, 2017 ▸). The SPI, FXS and FTH interfaces act as wrappers for a common experiment class, recognizing that CXDI experiments are at their core very similar, differing only in the number, type and relative position(s) of particle(s) in the beam [Fig. 2 ▸(*a*)]. For each shot, the particle or set of particles is randomly displaced and oriented in the volume intersected by the sample-delivery jet and beam, and the coherent diffraction pattern is computed [Fig. 2 ▸(*b*)]. The incorporation of random displacements accounts for the variable distance between the particles and detector that can result from aerosol injection. To facilitate incremental testing of reconstruction algorithms, either the ideal intensities or quantized photons can be saved, in addition to the number of particles, particle orientations and particle positions at each shot. Beyond Poisson error, the additional sources of noise described above can be defined in configuring the experiment and individually tuned to achieve the desired signal-to-noise ratio (Table 1 ▸).

## 3. Benchmarking

*Skopi* achieves rapid and high-throughput simulations of CXDI experiments through graphical processing unit (GPU) acceleration and parallelization using a message passing interface (MPI). For a reciprocal-space grid of fixed dimensions, the diffraction calculation is performed by direct summation over each atom’s contribution to the intensity and thus scales linearly with the number of atoms in the particle. Consequently, the full 3D diffraction volume for each particle is computed when an experiment is first configured and thus represents a one-time cost. Diffraction images are then efficiently calculated by slicing through this pre-computed volume at a rate independent of the number of atoms (Fig. S6). The resolution of this volume is determined by the detector distance and the X-ray wavelength, while its default dimensions are sufficiently large that interpolation error during slicing is negligible. The disadvantage of this approach is the inability to account for the stochastic effects of radiation damage during each interaction between the sample and beam (Nass, 2019 ▸); however, radiation damage is anticipated to be minimal for biological samples, particularly in comparison to instrumental background (Neutze *et al.*, 2000 ▸; Östlin *et al.*, 2019 ▸; Spence, 2017 ▸). On a single NVIDIA GeForce RTX 2080 ▸ Ti GPU, an SPI data set acquired on a one megapixel PnCCD detector could be simulated at a rate of 0.6 diffraction patterns per second after pre-computing the diffraction volume. This rate was reduced to 0.4 images per second for the SPI aggregate, FXS and FTH experiments due to the need to distribute clustered or dispersed particles in the beam for each shot. Data set generation can be easily parallelized across an arbitrary number of GPU nodes to increase throughput.

## 4. Validation

**Figure 3:**
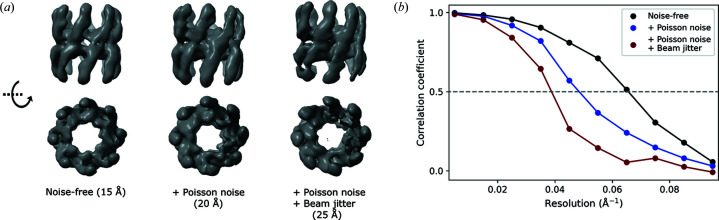
Reconstruction from simulated SPI data sets. SPI data sets from a chaperonin were simulated in the absence or presence of noise. A multi-tiered iterative phasing algorithm was used to recover the protein structure from 5000 images of the indicated data set. (*a*) Isosurfaces of the density map reveal the loss of eightfold symmetry with increasing noise. The resolution of each reconstruction is noted in parentheses. (*b*) The resolution was measured as the spatial frequency at which the FSC between the reconstructed and reference maps dropped to 0.5.

Comparison of SPI diffraction images simulated using *Skopi* and another CXDI software package, *Condor*, show that the two yield identical results within error from Poisson noise (Fig. S7). We then validated *Skopi* by recovering the protein structure from simulated diffraction images of a chaperonin (PDB 3iyf). SPI data sets consisting of 5000 images each were generated in the absence or presence of Poisson noise on a PnCCD detector positioned for a resolution limit of 14 Å at the edge of the detector. On average, the noisy data sets featured 0.0076 photons per pixel at the edge. The theoretical number of snapshots required for reconstructing a 70 Å particle at 14 Å resolution at this signal level is 3380 (Poudyal *et al.*, 2020 ▸; Ekeberg *et al.*, 2015 ▸). Reconstruction was performed using a Cartesian implementation of the multi-tiered iterative phasing (*MTIP*) algorithm (Donatelli *et al.*, 2017 ▸; Dujardin *et al.*, 2020 ▸). The resolution of each reconstruction was assessed by computing the Fourier shell correlation (FSC) between the recovered density and the ground-truth map,

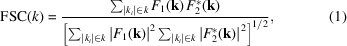

where *F*_1_ and *F*_2_ are the Fourier components of the density maps being compared and * denotes the complex conjugate. When compared with a noise-free reference map, the spatial frequency *k* at which the FSC falls to 0.5 is considered to estimate the resolution of the reconstruction (Liao & Frank, 2010 ▸). The protein structure was accurately recovered from noise-free diffraction images simulated on a PnCCD detector (Fig. 3 ▸), with a data set size comparable to what has previously been used to evaluate the *MTIP* algorithm (Chang *et al.*, 2021 ▸). As expected, the addition of Poisson noise degraded the quality of the recovered structure (Donatelli *et al.*, 2017 ▸), reducing the resolution from 15 to 20 Å. Introducing beam jitter further diminished reconstruction quality, as evident in the loss of the protein’s eightfold symmetry (Fig. 3 ▸). *Skopi* thus provides a useful tool to assess the tolerance of reconstruction algorithms to different types of noise.

## 5. Conclusions

We have presented *Skopi* as a convenient tool for the efficient simulation of CXDI experiments of noncrystalline biological samples. The modular design of this package makes it easy to represent each component of an XFEL experiment with a range of complexity, including modeling the most up-to-date features of current LCLS detectors (van Driel *et al.*, 2020 ▸). Another focus of *Skopi* is to produce simulated data with realistic noise. The different sources of error that typify CXDI experiments – including fluence jitter, beam miscentering and fluctuating dark noise – can be readily incorporated into the simulation without additional scripting.

The paucity of experimental CXDI data sets of non­crystalline biological samples has hindered algorithmic growth in the field. We anticipate that *Skopi* could help fill this gap through its capability of rapidly providing diverse simulated data sets with realistic noise. This may prove particularly valuable for the development of machine learning algorithms to perform classification, since labeled data can easily be generated in bulk. Classification algorithms would benefit data pre-processing to separate single-particle hits from aggregate shots and better assess hit rates, and could also be used during reconstruction to sort diffraction images based on the particle’s conformation (Ignatenko *et al.*, 2021 ▸), as done in cryo-electron microscopy (Punjani & Fleet, 2021 ▸; Zhong *et al.*, 2021 ▸; Chen & Ludtke, 2021 ▸).

In addition to aiding the development of reconstruction algorithms, we envisage *Skopi* as a potential tool for experimental design. By supporting both the advanced detectors currently in use at LCLS and the inclusion of fluctuating dark noise from past experiments, *Skopi* enables the simulation of highly realistic diffraction patterns that might be obtained at this facility. These simulated data sets, in turn, provide an estimate of the available signal under different experimental conditions, potentially allowing settings to be optimized and offering an estimate of the volume of data required for reconstruction in advance of XFEL experiments. The use of the *simS2E* software package to anticipate data collection needs at the European XFEL offers a precedent for optimizing CXDI experiments using simulation (Yoon *et al.*, 2016 ▸). Looking ahead, we are working towards making *Skopi* available within the larger *SIMEX* framework to facilitate large-scale start-to-end simulations of noncrystalline CXDI experiments (Fortmann-Grote *et al.*, 2017 ▸). In these frameworks, simulations are chained together starting from source parameters and progressing to propagation of the coherent X-rays through beamline optics, interaction of the photons with matter, and their subsequent detection and analysis. Such large-scale start-to-end simulations will be valuable for the development of noncrystalline XFEL experiments.

## 6. Code availability

*Skopi* is available through the Python Package Index (PyPi) at https://pypi.org/project/skopi/. The version referenced in this work is 0.5.1. More information about installation can be found at https://github.com/chuckie82/skopi/blob/main/docs/installation.md and additional example usage scripts at https://github.com/chuckie82/skopi/tree/main/examples/scripts.

## Supplementary Material

Additional figures. DOI: 10.1107/S1600576722005994/yr5084sup1.pdf

## Figures and Tables

**Table 1:** Sources of noise that can be modeled with *Skopi*

Type	Origin	Implementation
Poisson noise	Counting statistics	Quantization of diffraction intensities such that photon counting follows a Poisson distribution [equation (9)]
Aggregation	Sample delivery	Multi-particle clusters are generated using a ballistic aggregation model, with each particle randomly oriented and positioned with respect to the others
Heterogeneity	Sample dependent	A library of conformational states is generated by sampling along the particle’s normal modes
Hydration layer	Sample delivery	A solvent shell that follows the particle’s contours is represented using a continuum water model and is discretized to calculate the solvent contribution to diffraction (Liu *et al.*, 2012 ▸)
Beam miscentering	Beam characteristics	Displacements in the direct beam position relative to the detector center are assumed to be independent along each axis of the detector and Gaussian distributed
Fluence jitter	Beam characteristics	Shot-to-shot variation in the beam fluence is drawn from a Gaussian distribution
Fluctuating dark noise	Detector characteristics	Representative pedestal-subtracted dark shots from past LCLS experiments contribute incoherently
Static background	Parasitic scattering	The contribution from a custom background model is added incoherently to the diffraction intensities

## Appendix A Theory, implementation and usage

### A1. Elements of theory

In this section, we summarize the main elements of coherent X-ray optics taken from Paganin (2006 ▸) that allow us to derive the diffraction model implemented in *Skopi*. Propagation of electromagnetic waves Ψ(**r**, *t*) in non-magnetic matter follows the wave equation, where the local velocity is modulated by the local electric permittivity ε while the magnetic permeability μ_0_ is assumed to be that of free space everywhere,

Separation of temporal and spatial variables is assumed through spectral decomposition,

where the frequency ω of each monochromatic wave is related to its wavevector modulus *k* through the speed of light in free space, ω = *kc*. The effect of local perturbations can be factored in the refractive index *n*_ω_(**r**) = *c*[ε(**r**)μ_0_]^1/2^, which measures the relative speed of light in the medium compared with free space. Inserting the spectral decomposition of the electromagnetic wave into the wave equation yields an inhomogeneous Helmholtz equation for each monochromatic wave,

In free space, equation (4) simplifies to the homogeneous Helmholtz equation, which can be solved and yields the following solution to the wave equation (2):  =  . The spatial wavelength λ of this wave is such that  , so the wavevector modulus is related to it through the relation *k* = 2π/λ.

More generally, it can be shown that equation (2) is equivalent to its integral equation formulation, which involves the incident wave ϕ^(0)^ and a convolution with Green’s function  ,

In the Born approximation, the wavefunction on the right-hand side of equation (5) is assumed to be identical to the incident wavefunction, thus trivializing the integral equation formulation. Further assuming the case of Fraunhofer (far-field) diffraction and of an incident plane wave   , we can introduce the scattering amplitude *f*(Δ**k**), where Δ**k**  *k*(**r**/*r*) − **k**_0_,

Assuming a specific relationship between the refractive index and the electron density of the object ρ, the scattering amplitude is scaled by the Thomson scattering length *r*_0_,

Finally, denoting by 2θ the angle between the incident and resulting wavevectors, we introduce the notation **s** = Δ**k**, where  . In the superposition approximation, the molecular electron density is approximated as a sum of individual atomic contributions. In diffraction space, the atomic form factors *f*_*i*_ are tabulated as a function of  ,

The diffracted intensity *I* is scaled by the number of incident photons per beam focus area, or fluence *F*, which can be modeled as a constant or varying shot to shot. For a given detector pixel, the signal will be scaled by the solid angle Ω from the interaction point. The non-corrected diffracted intensity at pixel *i* thus reads *I*_*i*_ = *F*Ω_*i*_|*f*(**s**_*i*_)|^2^. However, polarization **u**_p_ of the incoming beam will result in non-isotropic Thomson scattering which further weights the intensity by |**u**_*i*_ × **u**_p_|^2^, where **u**_*i*_ is the unit vector pointing to pixel *i*. Optionally, to account for the multiple-spike nature of the X-ray beam spectrum due to SASE, the diffracted intensity is incoherently summed over a discrete set of varying frequencies weighted by the frequency content *w*_ω_. It is also possible to add different types of corrupting terms incoherently to the diffracted intensity, such as static noise or a sloped background.

Finally, encompassing the various terms listed above into functions α and β, the diffracted intensity is quantized by drawing from the corresponding Poisson distribution  to simulate the photon counts *C* measured on each pixel *i* of the detector,

### A2. Implementation

In practice, equation (8) is pre-computed on a discrete 3D cubic grid whose reciprocal extent is determined by the detector and beam parameters. Once the resulting diffraction volume is available in memory, it can be readily sliced at an arbitrary orientation and curvature in two steps. First, the detector pixels are mapped to their corresponding positions in the reciprocal volume. Second, the complex structure factor at each pixel is estimated through trilinear interpolation. The resulting diffraction intensities are then adjusted to account for beam polarization, solid-angle distortion and diverse sources of noise.

### A3. Usage

Python Script 1 illustrates how the modularity of *Skopi* is implemented in practice. Classes for the beam, particle and detector are instantiated and given to the experiment class, which in turn generates diffraction patterns. In the first example, files from a prior LCLS experiment are used to define the diffraction geometry. In general, a geometry file is not required.

Script 2 illustrates how to generate FXS images from five chaperone particles on a simple square detector.

The outputs from the two code examples above for SPI and FXS are shown in Fig. S8.
