# Supplementary material for: Skopi: a simulation package for diffractive imaging of noncrystalline biomolecules
Source: J Appl Crystallogr. 2022 Jul 15;55(Pt 4):1002–10. doi: 10.1107/S1600576722005994 (PMC9348890; doi:10.1107/S1600576722005994)
Supplement: Supplementary file 1 [file j-55-01002-sup1.pdf]

JOURNAL OF APPLIED CRYSTALLOGRAPHY

Volume 55 (2022)

Supporting information for article:

Skopi: a simulation package for diffractive imaging of noncrystalline biomolecules

Ariana Peck, Hsing-Yin Chang, Antoine Dujardin, Deeban Ramalingam, Monarin Uervirojnangkoorn, Zhaoyou Wang, Adrian Mancuso, Frédéric Poitevin and Chun Hong Yoon

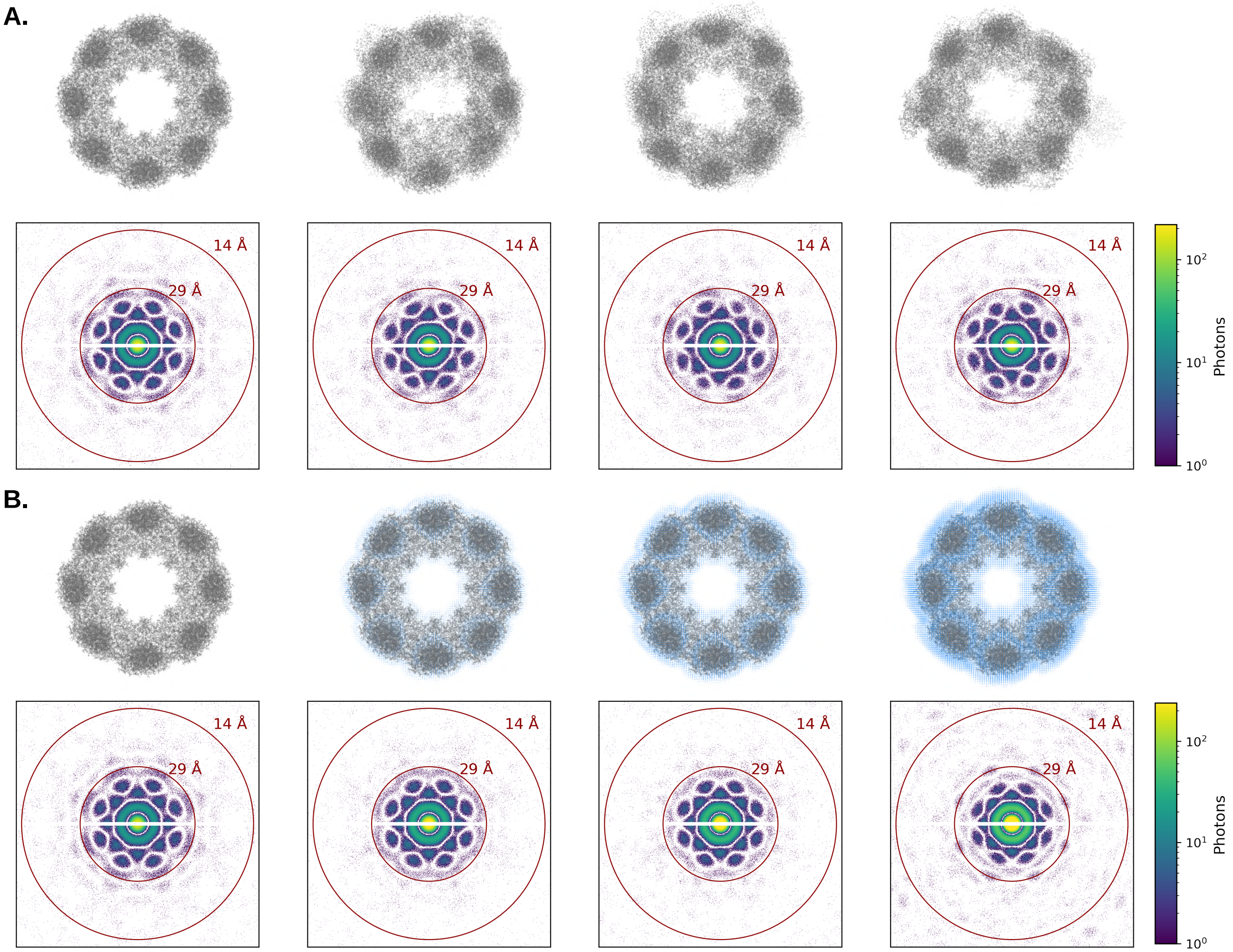

Figure S1

Sources of sample heterogeneity. (A) Distinct conformations were generated by sampling along the protein's normal modes (*upper*), and the corresponding diffraction patterns are visualized (*lower*). Conformational heterogeneity results in visible distortion of the protein's eight-fold symmetry along this axis in the simulated shots. (B) The hydration layer surrounding aerosolized particles is modeled as a disordered solvent shell of variable width that follows the contours of the protein. From left to right, the chaperonin protein (grey, PDB ID: 3iyf) was encased in a solvent shell (blue) of 0, 4, 8, and 12 Å (*upper*). The diffraction pattern for each solvation state is shown (*lower*).

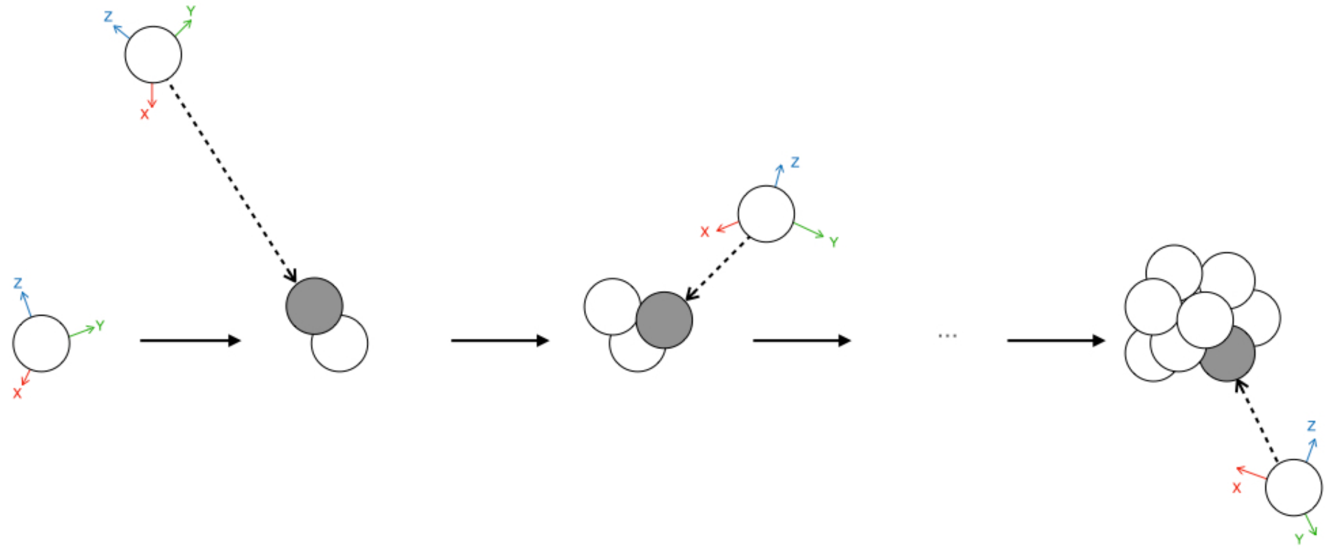

Figure S2

Particle aggregation. Schematic description of BPCA model where filled circles represent particles newly added to the aggregate. At each step, a new particle is randomly oriented and positioned and then translated towards the cluster till it hits and sticks.

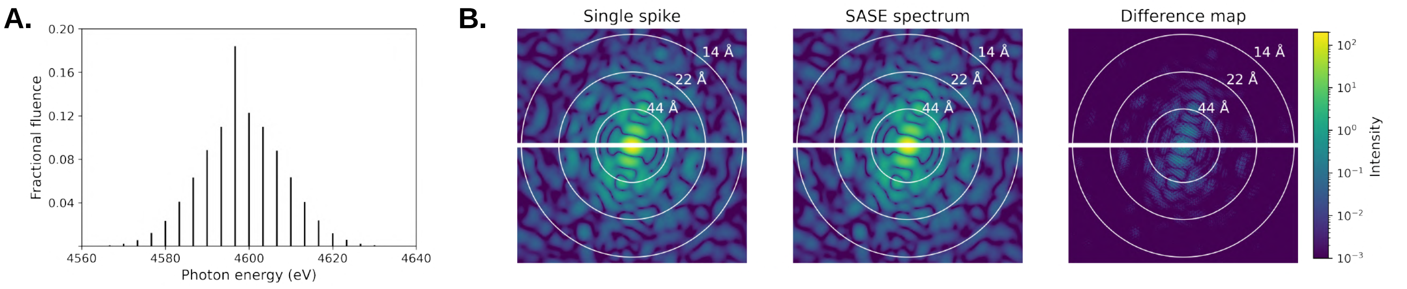

Figure S3

Modeling the beam as a SASE spectrum. (A) The distribution of spike energies and fractional fluences in a representative SASE spectrum are shown for a mean beam energy of 4600 eV. (B) Diffraction patterns for a particle in the same orientation but imaged with either a single monochromatic beam or a SASE-style beam are compared, with the absolute difference map shown on the right. Because the magnitude of differences is on the same scale as quantization error, intensities rather than photons were computed. However, the source of the difference does not follow a Poisson distribution.

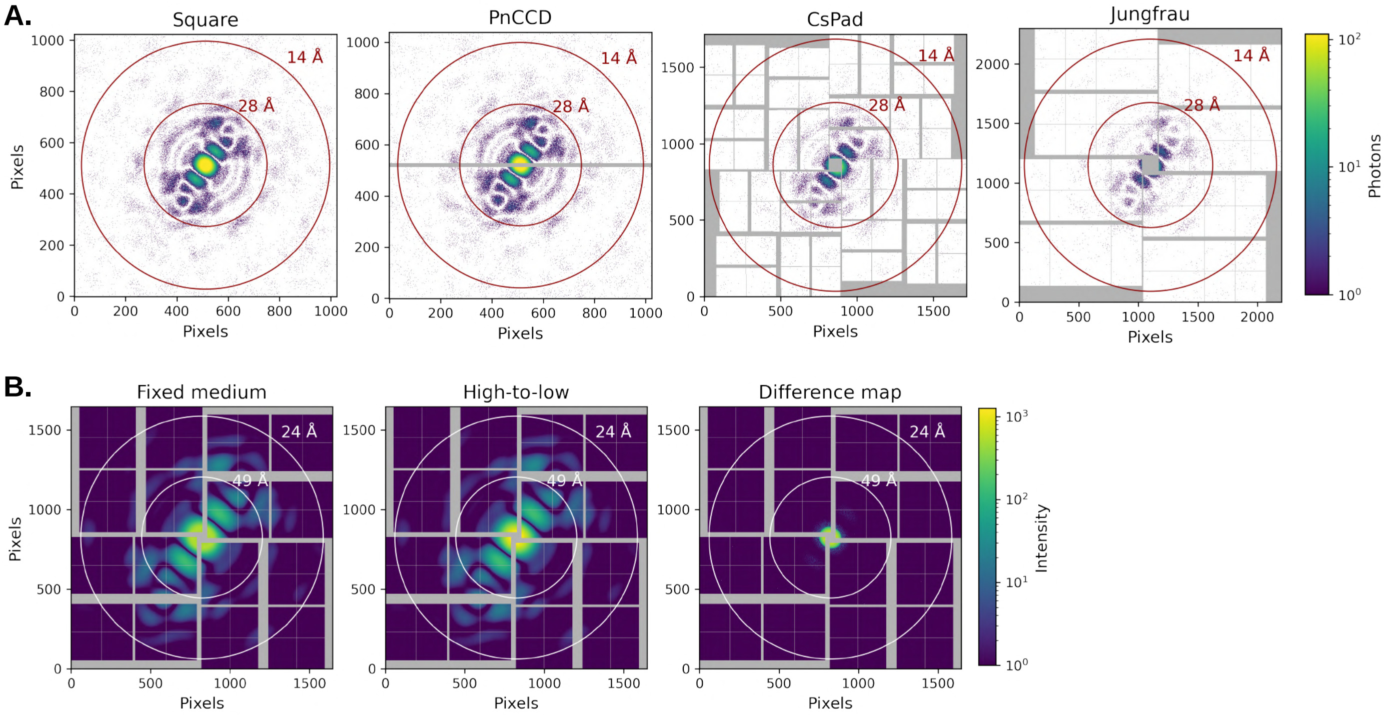

Figure S4

Support of diverse detector types. (A) In addition to a user-defined monolithic square detector, *skopi* supports simulating images on a range of LCLS detectors such as the PnCCD, CsPad, and Jungfrau. Diffraction patterns using the same beam and particle objects were simulated on each of these detectors. (The photon counts appear dissimilar between the patterns due to the detectors' different pixel surface areas, but the integrated counts are roughly the same after accounting for the gaps between detector panels.) (B) The recently-developed Jungfrau and Epix10k detectors have an auto-ranging feature with various gain combinations to increase the dynamic range. Diffraction patterns simulated on the Epix10k are compared for the fixed medium and high-to-low gain combinations, showing that the latter mode eliminates saturation at high intensity values near the detector center. Simulations could guide which gain mode setting to use prior to experiments especially for inorganic materials where diffracted intensities can be intense. Intensities rather than photons are shown and the detector distance has been adjusted to better illustrate where differences between the gain combination modes are most pronounced.

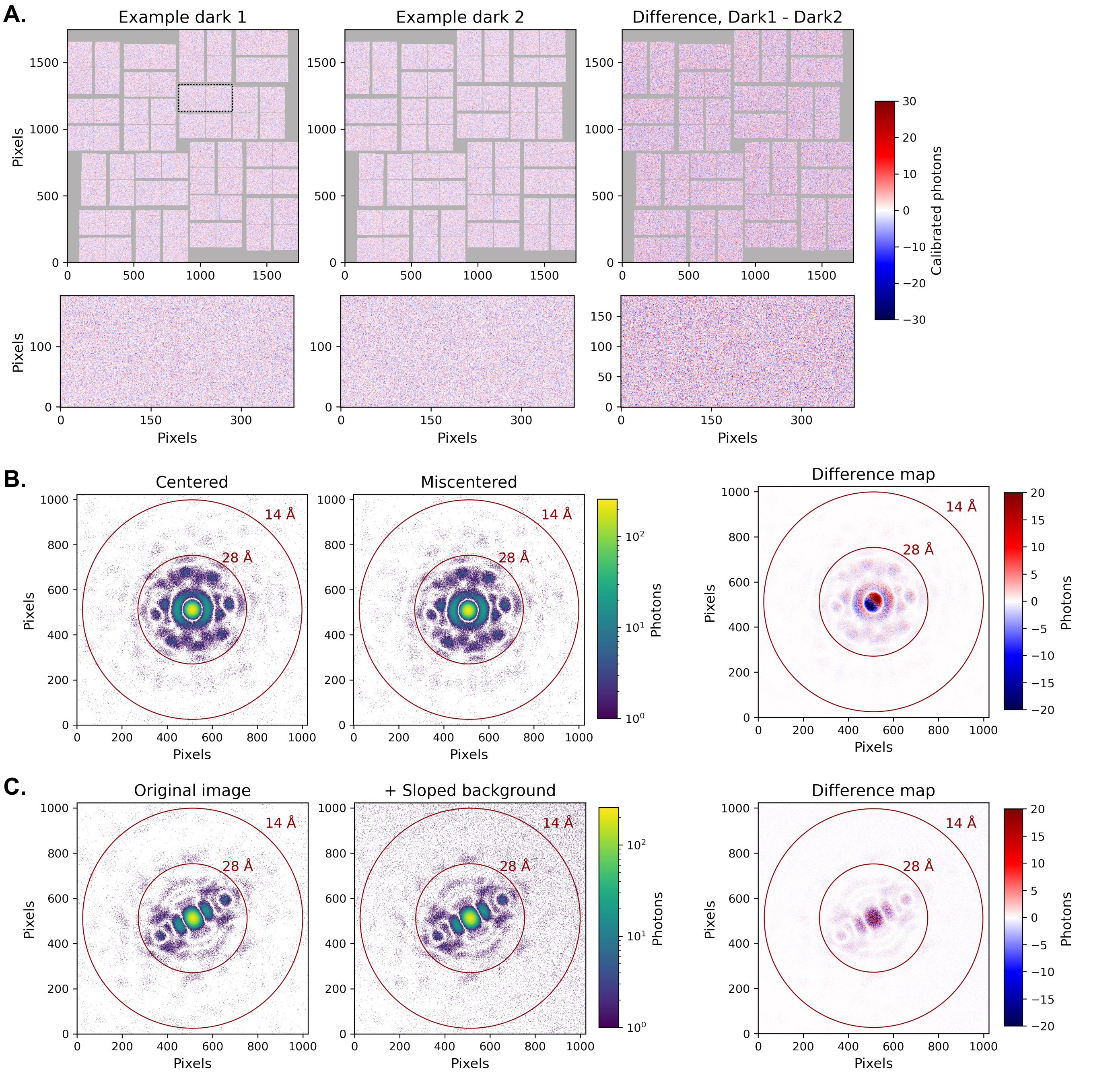

Figure S5

Sources of instrument noise. (A) Representative dark shots and their difference image are shown from an experiment performed at LCLS on a CsPad detector (*upper*). The dark noise fluctuates between images, and the counts can be negative due to pedestal subtraction. The detector panel outlined in black is shown in greater detail below each image to better visualize the unstructured nature of dark noise (*lower*). (B) Beam miscentering and (C) a user-defined sloped background can be introduced as additional sources of noise. Difference maps are shown on a linear scale to aid visual comparison.

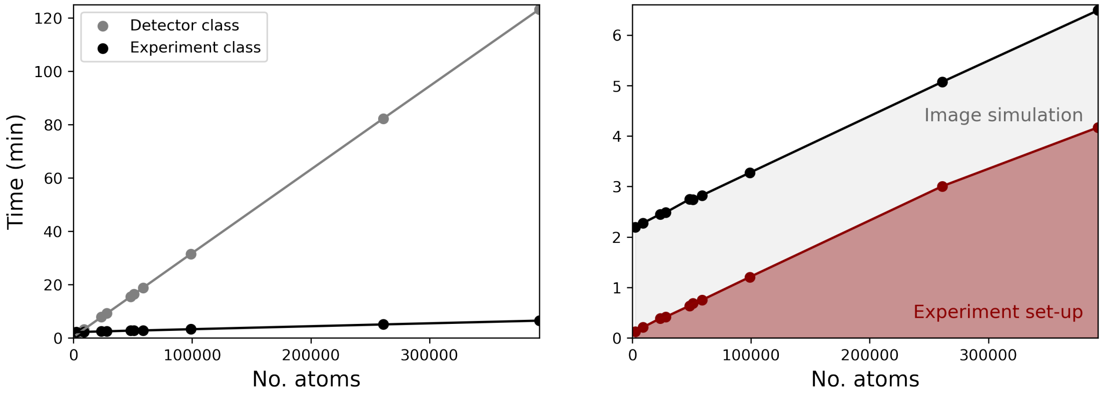

Figure S6

Scaling of the diffraction calculation with particle size. Diffraction images can be simulated through either the Detector or Experiment classes. The time required to simulate 100 SPI images on a PnCCD detector using the two different approaches is compared (*left*). The Experiment class accelerates image simulation by pre-computing the 3D diffraction volume, a step that scales linearly with the number of atoms in the particle (*right, red*). Diffraction images are then calculated by slicing through this volume, such that the compute time is constant as a function of particle size once the Experiment class is set up (*right, grey*). Calculations were performed on an NVIDIA GeForce RTX 2080 Ti GPU; the PDB IDs of the particles assessed were: 1efr, 1i3q, 2axt, 2cex, 3cc4, 3gpw, 3iyf, and 4k5y.

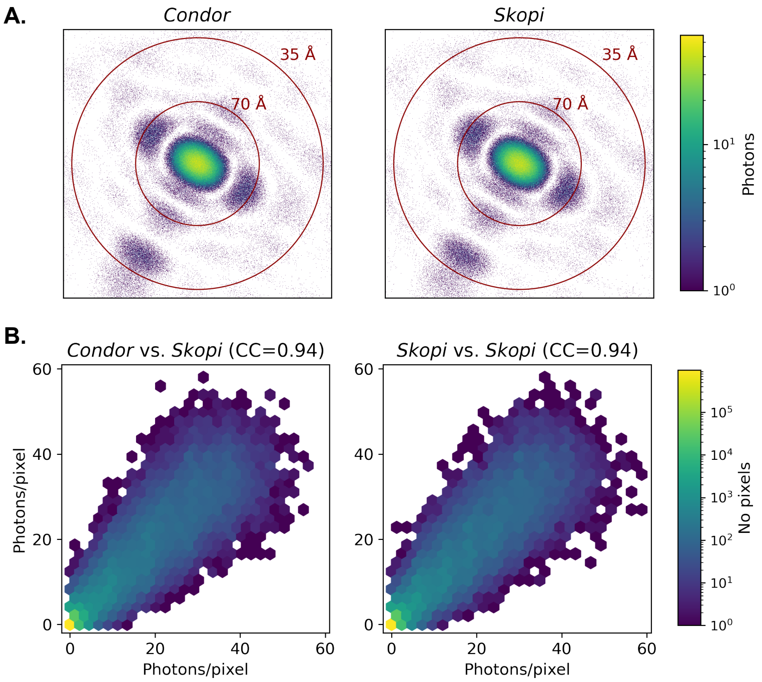

Figure S7

Comparison to the *Condor* software package. (A) Diffraction images of an identically-oriented chaperonin protein (PDB ID: 3iyf) simulated using the same SPI experimental parameters by the indicated software package are compared. (B) The photon counts from corresponding pixels between the images in (A) are compared using hexbin plots (*left*). For reference, two independent diffraction images generated by *skopi* using the same experimental parameters are also compared (*right*), indicating that the difference between the *Condor* and *Skopi* images is due to Poisson noise. The overall correlation coefficient between pairs of images is noted in parenthesis.

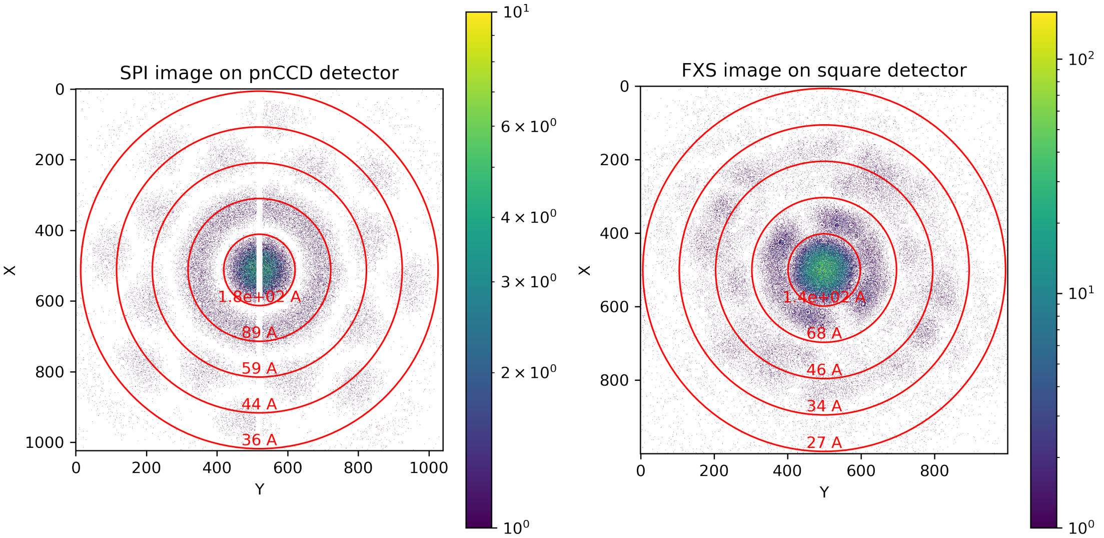

Figure S8

Diffraction patterns from example code. SPI image of a chaperonin (PDB ID: 3iyf) on a pnCCD detector (*left*) and FXS image from five chaperonin particles on a generic square detector (*right*). By default, the visualizer in *skopi* follows the coordinate conventions of matplotlib.
